# Neutralizing Antibody Response to SARS-CoV-2 Variants After Two mRNA COVID-19 Vaccine Doses in a Cohort of Patients with Inflammatory Bowel Disease from a Southern Italy Tertiary Hospital

**DOI:** 10.3390/healthcare13050508

**Published:** 2025-02-26

**Authors:** Dario Genovese, Daniele Brinch, Stefano Muscarella, Marica Saladino, Lucio Carrozza, Chiara Cunsolo, Giuseppa Luisa Sanfilippo, Emanuele Amodio, Maria Cappello, Donatella Ferraro

**Affiliations:** 1 Hygiene and Preventive Medicine Section, Department of Health Promotion Sciences, Maternal and Infant Care, Internal Medicine and Medical Specialties (PROMISE), University of Palermo, 90127 Palermo, Italy; 2 Gastroenterology and Hepatology Section, Department of Health Promotion Sciences, Maternal and Infant Care, Internal Medicine and Medical Specialties (PROMISE), University of Palermo, 90127 Palermo, Italy; 3 Microbiology Section, Department of Health Promotion Sciences, Maternal and Infant Care, Internal Medicine and Medical Specialties (PROMISE), University of Palermo, 90127 Palermo, Italy

**Keywords:** inflammatory bowel disease, COVID-19, SARS-CoV-2, neutralizing antibodies

## Abstract

**Introduction:** Inflammatory bowel diseases (IBDs) require immunosuppressive drugs like biologics. All IBD patients, including those on biological therapy, should be vaccinated against COVID-19, according to the ECCO recommendations. IBD patients on anti-TNF treatment exhibited lower COVID-19 vaccine responses; however, SARS-CoV-2 variant neutralizing antibody titers have been seldom studied. **Methods:** IBD patients and healthcare professionals (control group) were tested for COVID-19 vaccine immunogenicity by neutralizing antibody titers against Wild-Type SARS-CoV-2 and its variants. IBD patients were assigned to no treatment/mesalamine, anti-TNF biologic therapy, or non-anti-TNF biologic therapy. The study was performed in a tertiary hospital in Palermo, Sicily, from May to July 2021. **Results:** In total, 107 IBD patients and 41 healthcare workers were enrolled. A total of 46 patients received mesalamine or no medication, 28 received anti-TNF biologics, and 33 received non-anti-TNF biologics. No significant differences were found in age, gender, or timing of blood sampling post vaccination. Omicron neutralizing activity was markedly reduced in all groups (*p* < 0.001). The group of patients on anti-TNF biologics showed lower neutralizing antibody titers against Alpha, Delta, and Gamma strains than every other group analyzed. **Conclusions:** IBD patients on anti-TNF drugs have a reduced serological response to the SARS-CoV-2 vaccine, with the Omicron variant not being neutralized. This highlights the necessity for tailored vaccine strategies for these patients.

## 1. Introduction

Since the outbreak of COVID-19, numerous SARS-CoV-2 variants have been identified. In late 2020, Alpha, Beta, and Gamma variants emerged, followed by the Delta strain in January 2021 and Omicron in November 2021 as the last variants from that period. These SARS-CoV-2 strains were categorized as variants of concern (VOCs) due to their increase in transmission and a substantial decline in neutralization by antibodies generated by either previous infection or immunization [1,2,3,4].

A recent review article demonstrated that neutralizing antibodies play a crucial role in long-term immunity by consistently targeting and reducing infections when they are encountered a second time [5]. Following a first infection or immunization, the immune system stores a recollection of the pathogen, generating neutralizing antibodies that can promptly react to subsequent infections. These antibodies attach to specific viral proteins, which stop the virus from entering host cells and consequently prevent the infection. The persistent circulation of neutralizing antibodies in the bloodstream and their capacity to deliver prompt and efficient responses are shown to be vital for maintaining immunity over a longer period [5].

The ability of SARS-CoV-2 vaccines to neutralize these variants has been a focal point of research. SARS-CoV-2 vaccines indeed alter the natural course of the disease, as indicated by high serological conversion rates in the general population. Vilibic-Cavlek et al. [6] found that neutralizing antibodies produced both after vaccination or natural infection exhibit significant effectiveness against different SARS-CoV-2 variants, such as Alpha, Delta, and Omicron. Although neutralizing antibodies produced only from vaccination were effective, those who received both vaccination and subsequent infection showed the highest level of neutralizing activity against different strains.

The present study focused on inflammatory bowel diseases (IBDs), which are immuno-mediated diseases that may necessitate the use of immunosuppressive drugs or biological therapy for treatment. Patients treated with biological therapies are considered to be immunocompromised and may be at increased risk of infection and severe sequalae due to the COVID-19 [7]. Moreover, individuals with an inflammatory bowel disease are considered a population at increased risk of vaccine-preventable diseases [8]. It is for these reasons that the European Crohn’s and Colitis Organization (ECCO) strongly recommends vaccinating all IBD patients at diagnosis and before starting immunosuppressive and/or biological therapy against specific viruses, mycobacteria, bacteria, fungi, and parasites, including COVID 19 infection [9].

Some studies have evaluated the serological response in patients with IBD but only a few have studied the neutralizing activity towards each SARS-CoV-2 variant [10,11,12,13]. Therefore, our study sought to examine the neutralizing antibody titers against SARS-CoV-2 variants among patients with an IBD and different treatment regimens, comparing their neutralizing activity to that of a control group composed of healthcare professionals working in the tertiary-level hospital in which the study took place.

This study expands upon prior research by specifically analyzing the neutralizing antibody titers against different SARS-CoV-2 variants in IBD patients undergoing distinct treatment regimens. While previous studies have demonstrated a reduced immune response in IBD patients treated with anti-TNF therapy, few have comprehensively compared neutralizing activity across multiple VOCs in this population. Our findings reinforce the evidence of diminished NtAb titers against Omicron in all groups, with the most significant reduction observed in patients receiving anti-TNF therapy. This underscores the need for tailored vaccination strategies, particularly for at-risk individuals, to enhance immune protection against emerging SARS-CoV-2 variants.

## 2. Materials and Methods

### 2.1. Population

This study is a prospective monocentric case–control study conducted at the Policlinico University Hospital “P. Giaccone” of Palermo, a tertiary-level hospital. Consecutive IBD patients were recruited, and hematological sera were collected between May and July 2021 and thereafter analyzed.

The study population was divided into four demographic groups:

- Patients with IBD who receive biological anti-TNF therapy for at least 6 weeks (infliximab, adalimumab, golimumab).
- Patients with IBD undergoing non-anti-TNF biological therapy for at least 6 weeks (vedolizumab, ustekinumab).
- Patients with IBD treated with mesalamine for at least 12 weeks, or untreated.
- A sample of healthcare professionals of the tertiary hospital.

Inclusion criteria were the following:

- For the first three groups: to have an established diagnosis of Crohn’s disease (CD) or Ulcerative Colitis according to ECCO guidelines for UC and CD.
- To be aged 18 years or older.
- To have received solely two doses of mRNA COVID-19 vaccines (BNT162b2 or mRNA-1273).

Healthcare professionals with no systemic autoimmune or inflammatory bowel diseases were recruited to represent the control group. 

This study was approved on 21 June 2021 by the Ethics Committee of A.O.U.P. University Hospital of Palermo “P. Giaccone”, Palermo (ID code number 6). Each participant provided written informed consent. Blood samples were drawn between days 14 and 65 after the second vaccine dose and clinical and demographic characteristics were recorded.

All the subjects with a documented SARS-CoV-2 infection before the recruitment phase or affected by a primary immunodeficiency or human immunodeficiency virus infection were excluded.

The vaccine was administered to the healthcare workers and patients according to the schedule established by the Italian National Vaccination Plan. Demographic data, smoking habits, body mass index, co-morbidities, clinical disease features and activity, current drugs, and laboratory tests (hemoglobin, white blood cells, C-reactive protein, albumin) were registered at the time of SARS-CoV-2 vaccination.

Vaccine safety was assessed through the incidence of reported adverse events during the 15 days following each vaccine dose administration.

### 2.2. Neutralizing Antibodies

To assess neutralizing antibodies against Wild-Type SARS-CoV-2 and its VOCs, specifically Alpha, Delta, Gamma, and Omicron, collected from clinical samples, we used a previously described in vitro live virus neutralization assay. All live virus micro-neutralization assays were carried out in a BioSafety Level 3 (BSL-3) laboratory. Clinical specimen testing methods were performed in strict accordance with WHO interim guidance. Neutralizing antibody (NtAb) titers were evaluated utilizing Vero E6 cells infected with Wild-Type virus and each VOC individually. NtAb titers were defined as the reciprocal value of the sample dilution that provided 50% protection against virus-induced cytotoxicity (ID50). Titers with a value less than 10 were classified as “negative”.

### 2.3. Statistical Analysis

Continuous variables were shown as mean (standard deviation—SD) if normally distributed and median (interquartile range—IQR) if not normally distributed. Categorical variables were summarized by frequency and relative frequencies (%), whereas continuous variables were shown as mean (standard deviation—SD) if normally distributed and median (interquartile range—IQR) if not normally distributed. Furthermore, from the international scientific literature, neutralizing antibody titers were summarized by geometric mean and geometric standard deviation (GSD). The Shapiro–Wilk normality test was used to determine the normal distribution of continuous data. Pearson’s Chi-squared test was used to compare categorical data between groups, while non-parametric continuous variables were compared using the Kruskal–Wallis test. The Mann–Whitney U-test was used to investigate inter-group comparisons. All statistical analyses were carried out using R for Statistical Computing (version 4.2.2, Vienna, Austria) within the RStudio interface (RStudio, PBC, Boston, MA, USA), with a *p*-value of 0.05 deemed statistically significant.

## 3. Results

Overall, the study sample was composed of 148 participants, 107 of which suffered from an inflammatory bowel disease (IBD), while the remaining 41 were health professionals representing the control group. The main demographic characteristics are presented in Table 1.

At the time of the recruitment, the IBD group was receiving the following treatments:

- A total of 46 patients were undergoing either conventional therapy (mesalamine) or no therapy.
- A total of 28 patients had received anti-TNF biological drugs (24, infliximab; 3, adalimumab; and 1 patient under golimumab treatment).
- A total of 33 patients were treated with non-anti-TNF biological drugs (5, ustekinumab; 28, vedolizumab).

The median age of included participants was between 46 years (in the biologic anti-TNF drug group; IQR: 37–63) and 56 years (among those who underwent conventional treatment; IQR: 49.25–64). The F:M ratio was between 1.36 (in the biological non-anti-TNF drug group) and 0.55 (in the biological anti-TNF drug group). There were no statistically significant differences either for median age or for the distribution between men and women within each group investigated; furthermore, no difference was found in the interval between the second mRNA COVID-19 vaccine dose and the serological sample.

As reported in Table 2, roughly half of the patients included suffered from Ulcerative Colitis (55 patients out of 107, 51.4%) with slight and non-statistically significant differences in the distribution between the three groups. No statistically significant differences were evidenced for almost all the investigated variables but smoking habits, ESR, and CRP. As per the smoking habits, most of the non-smokers belonged to the biologic non-anti-TNF drug group (24 patients, constituting 72.8% of the considered group), whereas the other two groups had a higher percentage of smokers and former smokers (*p* = 0.007). The hematological serum samples allowed us to evaluate two of the widest-used biomarkers of inflammation: erythrocyte sedimentation rate (ESR) and C-reactive protein (CRP). Both the levels were either statistically different or tended towards a significant difference between the considered groups, with higher levels among the subjects being treated with biological non-anti-TNF drugs.

The general serological features of the four population groups are shown in Table 3. The geometric mean NtAbs titers in the control group range from 9.59 (±2.07) against the Omicron strain to 49.03 (±2.58) against Gamma. In the conventional treatment group, they range from 9.56 (±1.91) against Omicron to 39.82 (±2.22) against the Delta strain; in the biologic anti-TNF drug group, from 7.52 (±1.81) against Omicron to 27.52 (±3.11) against Gamma. Finally, in the biologic non-anti-TNF drug group, the NtAbs titers range from 8.64 (±1.81) against Omicron strain to 38.23 (±2.13) against Delta variant. As per the absence of NtAbs against the evaluated variants, the Omicron strain was the variant for which the NtAbs titers were more frequently undetectable: 64% of the biologic anti-TNF drug group could not respond adequately against Omicron, as well as approximately 50% of the remaining three groups.

**Figure 1:**
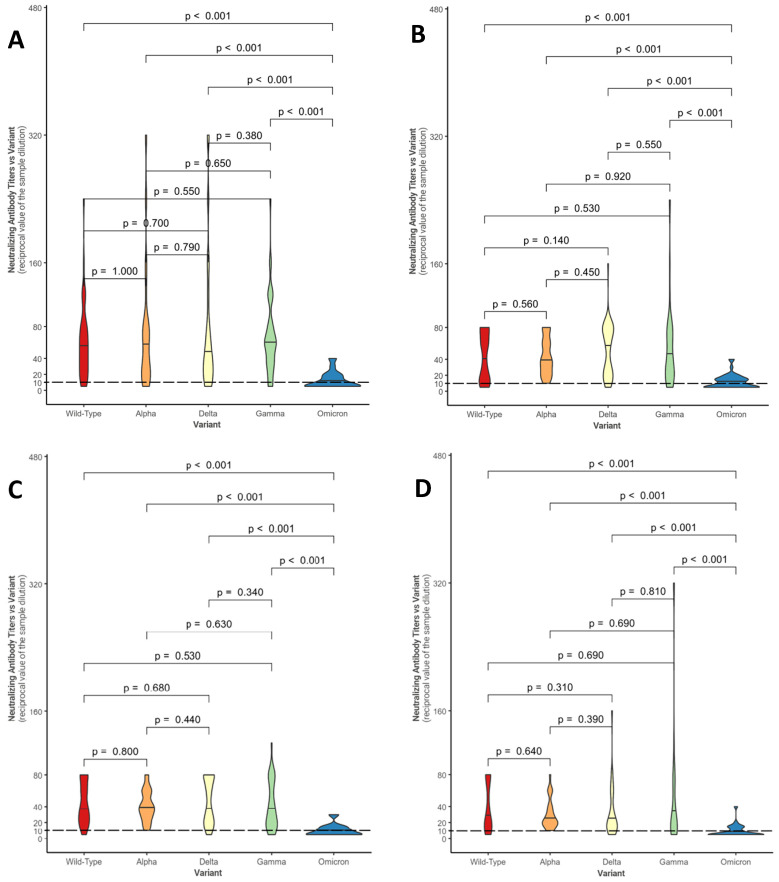
Comparison between neutralizing antibody titer distributions against the different variants within the control group (**A**), within the conventional treatment group (**B**), within the biologic anti-TNF drug group (**C**), and within the biologic non-anti-TNF drug group (**D**). The Mann–Whitney U-test was used with associated *p*-values. Titers below 10 (---) are considered negative.

Figure 1 shows, in the form of violin plots, the distributions of NtAbs titers against each considered variant for the four groups (control group—Figure 1A; conventional treatment—Figure 1B; biologic anti-TNF drugs—Figure 1C; biologic non-anti-TNF drugs—Figure 1D).

Overall, in each group, statistically significant differences were found between the median neutralizing activity against the Omicron variant and all the other SARS-CoV-2 variants (*p*-value < 0.001). These results confirm what has been evidenced in Table 3: in a large number of patients, neutralizing activity against Omicron is absent (in fact, 20/45 subjects in conventional therapy do not show a response, 17/27 subjects in anti-TNF and 16/34 under non-anti-TNF biological drugs; even in the control group, neutralizing activity is absent in 20/41 individuals).

**Figure 2:**
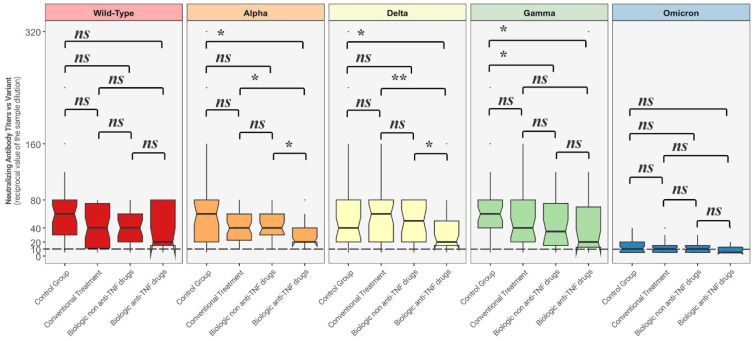
Comparison between neutralizing antibody titer distributions against each variant among each of the four groups. The Mann–Whitney U-test was used with associated *p*-values. Titers below 10 (---) are considered negative. ns = non-statistically significant; * = *p* < 0.05; ** = *p* < 0.01.

Figure 2 depicts comparisons between distributions of NtAbs titers in each group against all the different variants.

Neutralizing antibody titers against Alpha and Delta strains were statistically significantly lower among the patients undergoing biologic anti-TNF drugs when compared to the other considered groups, while NtAbs titers against the Gamma variant among those treated with biologic anti-TNF therapy and biologic non anti-TNF group were both statistically significantly lower than the ones produced by the control group.

## 4. Discussion

The main aim of this study was to evaluate the levels of neutralizing activity against different strains of SARS-CoV-2, including the ancestral strain and the main VOCs. Our study shows that after the second mRNA COVID-19 vaccine dose, the NtAb titers are comparatively significantly lower against the Omicron variant than against the other VOCs and Wild-Types, and this is valid for each group. This study found that neutralizing activity was achieved in each variant, except for Omicron, with significant differences between the groups. In fact, those with IBD who were receiving anti-TNF alpha therapy (predominantly infliximab) exhibited lower NtAb titers compared to both the control group and IBD patients receiving conventional therapy or non-anti-TNF biologics. These findings are consistent with the research study conducted by Dayam et al. [14], which aimed to assess the immune response directed towards SARS-CoV-2 in individuals diagnosed with immune-mediated inflammatory diseases, including those with inflammatory bowel disease (IBD), who were recruited for the study. The study looked at multiple aspects, including the neutralizing response to Wild-Type and several VOCs. The assessment was carried out at two separate time points: between 2 and 4 weeks after the second mRNA vaccine dose and in the 3rd month following the second dose. As a result, individuals who underwent therapy with anti-TNF biological medications exhibited a notably reduced neutralizing response in comparison to those who received different treatments or remained untreated. Moreover, during the third month, the neutralizing response directed towards the Omicron variant appeared to be absent in all the analyzed groups. Unfortunately, the aforementioned article did not consider the IBD participants per se.

Data from COVID-19 vaccinations reveal diminished immune response among IBD patients treated with anti-TNF and Tofacitinib [15,16,17]. To the best of our knowledge, there is limited scientific literature evaluating neutralizing antibody titers against SARS-CoV-2 variants in individuals with an inflammatory bowel disease, confronting them with healthy controls.

Two studies conducted by Edelman-Klapper and Rabinowitz’s research group [10,11] assessed the neutralizing activity towards Wild-Type SARS-CoV-2 in three different groups (namely the anti-TNF and non-anti-TNF groups, and a healthy control group), concluding that the NtAbs were significantly lower amongst those who were treated with anti-TNF biologics. Those with non-anti-TNF treatment had a comparable immune response with the control group. Some studies investigated the neutralizing response after three doses of vaccine towards several VOCs among patients with IBD. In this case, López-Marte et al. [12] demonstrated, in a limited sample size, that patients receiving anti-TNF treatment exhibited lower NtAb levels towards all VOCs, with a notable decrease in Omicron, in comparison to healthy controls. This partially differs from our study results, suggesting that the serological difference between individuals treated with anti-TNF and the general population becomes more pronounced, especially in the case of the Omicron variant, after the administration of the third vaccine dose. Similarly, Liu et al. [13] demonstrated that individuals treated with Tofacitinib and anti-TNF biologics exhibited lower NtAb titers compared to those treated with vedolizumab, particularly for the Omicron variant, raising some concerns about vaccine effectiveness among these patients. It is noteworthy to underline that after the third dose, an overall rise in the neutralizing activity has been documented.

It is remarkable to mention that this study has some limitations. The small sample size of participants and the fact that they were selected from a single tertiary hospital could affect the generalizability of the results to a broader population. Additionally, the absence of a formal sample size calculation, as observed in similar studies, may limit the statistical power and applicability of the findings. Moreover, we used antibody neutralization as a proxy of vaccine effectiveness although some patients could also become infected in the presence of neutralizing antibodies, or others, at least theoretically, could still be protected in absence of neutralizing antibodies because of cellular immunity.

The present study enriches existing research by meticulously comparing neutralizing antibody responses to specific SARS-CoV-2 variants in IBD patients undergoing different treatments—anti-TNF therapy, non-anti-TNF biologics, no therapy, or mesalamine—alongside a control group. Unlike prior studies that primarily examined the general population, our findings highlight a notable reduction in neutralizing activity among specific patient groups.

This finding lines up with recent research suggesting that vaccine effectiveness may be influenced by emerging SARS-CoV-2 variants, such as the JN.1 sub-lineage, which exhibit antigenic deviations from previous strains. A recent study indicated that reduced vaccine effectiveness has been noted during periods dominated by these variants, highlighting the necessity for continual improvements to COVID-19 vaccines to enhance protection especially in at-risk groups [18].

These results underscore the imperative for the meticulous monitoring of these patients. The bivalent COVID-19 vaccines demonstrated an improved capacity to elicit an antibody response against SARS-CoV-2 variants in comparison to their monovalent counterparts [19]. Nonetheless, the effectiveness of these vaccinations, especially in vulnerable groups like IBD patients undergoing anti-TNF treatment, may remain attenuated compared to the general population, hence underscoring the necessity for tailored vaccination strategies. In this regard, it is crucial to transition from a passive approach—where patients are expected to seek out vaccinations—to a proactive, patient-centered model that integrates vaccination into routine clinical care. This strategy should involve multidisciplinary collaboration among healthcare providers, including specialists in both hospital and community settings, to actively promote and facilitate vaccine administration during routine follow-up visits, hospitalizations, and outpatient care. Moreover, embedding vaccination recommendations within personalized treatment plans and leveraging digital health infrastructure to track immunization status could enhance accessibility and adherence, ultimately improving protection in at-risk populations.

Additional research is required to ascertain if the newly developed vaccines may enhance immunogenicity in specific population groups, including patients with inflammatory bowel disease, particularly those receiving anti-TNF biological therapy. Given the increasing evidence that vaccine-induced immunity may wane over time and exhibit reduced effectiveness against emerging variants [20], periodic vaccination updates and continuous surveillance are essential elements of prevention and control strategies for this vulnerable population.

## Figures and Tables

**Table 1:** Demographic characteristics of the control group and the three study groups: those receiving conventional therapy, those receiving anti-TNF therapy, and those receiving non-anti-TNF treatment. For the variable “*Sex*”, the Chi-square test was used. Regarding the variables “*Age*” and “*Days to serum collection from the 2nd mRNA vaccine dose*”, their normality was first assessed. After having verified that their distribution was not normal, the Kruskal–Wallis test was applied.

	Group, Median (IQR)				*p*-Value
	Control Group (*N* = 41)	Conventional Treatment (*N* = 46)	Biologic Anti-TNF Drugs (*N* = 28)	Biologic Non Anti-TNF Drugs (*N* = 33)	
***Sex, N (%)***					0.19
- **Females**	18 (43.9%)	16 (34.8%)	10 (35.7%)	19 (57.6%)	
- **Males**	23 (56.1%)	30 (65.2%)	18 (64.3%)	14 (42.4%)	
***Age in years***	54 (48–57)	56 (49.25–64)	46 (37–63)	51.5 (43–66)	0.28
***Days to serum collection from 2nd mRNA vaccine dose***	21 (17–45)	30 (22–44.5)	30 (22–38)	31.5 (24.25–46.25)	0.29

**Table 2:** Clinical and anamnestic characteristics of the three groups of patients with IBD.

	Conventional Treatment (*N* = 46)	Biologic Anti-TNF Drugs (*N* = 28)	Biologic Non Anti-TNF Drugs (*N* = 33)	*p*-Value
*IBD type, N (%)* *				0.65
- **Ulcerative Colitis**	26 (56.5%)	13 (46.4%)	16 (48.5%)	
- **Crohn’s Disease**	20 (43.5%)	15 (53.6%)	17 (51.5%)	
*Body Mass Index, mean (±SD)* *	24.8 (±3.6)	24.8 (±3.3)	25.6 (±3.5)	0.57
*Cardiovascular Diseases, N (%)* *	17 (37%)	6 (21.4%)	6 (18.2%)	0.13
*Diabetes, N (%)* *	4 (8.7%)	1 (3.6%)	3 (9.1%)	0.69
*COPD and/or Asthma, N (%)* *	1 (2.2%)	0 (0%)	2 (6.1%)	0.36
*Chronic Renal Failure, N (%)* *	0 (0%)	1 (3.6%)	2 (6.1%)	0.27
*Tumor, N (%)* *	3 (6.5%)	1 (3.6%)	5 (15.1%)	0.22
*Cirrhosis, N (%)* *	2 (4.4%)	0 (0%)	0 (0%)	0.26
*Smoking habits, N (%)* *				**0.015**
- **Non-smoker**	19 (41.3%)	9 (32.2%)	24 (72.8%)	
- **Smoker**	11 (23.9%)	6 (21.4%)	4 (12.1%)	
- **Former smoker**	16 (34.8%)	13 (46.4%)	5 (15.1%)	
***ESR (cut-off value = 1), median (IQR)* ^^^**	0.54 (0.25–0.8)	0.57 (0.32–1.03)	0.8 (0.65–1.34)	**0.007**
***CRP (cut-off value = 1), median (IQR)* ^^^**	0.22 (0.1–0.78)	0.2 (0.1–0.77)	0.68 (0.22–1.24)	0.059

* The Chi-square test or Fisher’s exact test was used, where appropriate. ^^^ The Kruskal–Wallis test was applied after verifying, using the Shapiro–Wilk test, that the distribution of each variable was not normal. Statistically significant *p*-values are highlighted in bold.

**Table 3:** Characteristics of the control group and the three study groups of patients with IBD in terms of neutralizing antibody titers and their absence per each variant analyzed.

	Control Group (*N* = 41)	Conventional Treatment (*N* = 46)	Biologic Anti-TNF Drugs (*N* = 28)	Biologic Non Anti-TNF Drugs (*N* = 33)
***Neutralizing Antibody titers against variants (reciprocal value of the sample dilution), geometric mean (±GSD)***				
- **Wild-Type**	45.11 (±2.37)	30.39 (±2.59)	26.87 (±2.66)	35.53 (±2.12)
- **Alpha**	45.15 (±2.94)	38.05 (±1.81)	25.75 (±1.79)	36.39 (±1.74)
- **Delta**	42.64 (±3.27)	39.82 (±2.22)	22.95 (±2.43)	38.23 (±2.13)
- **Gamma**	49.03 (±2.58)	35.61 (±2.66)	27.52 (±3.11)	31.44 (±2.31)
- **Omicron**	9.59 (±2.07)	9.56 (±1.91)	7.52 (±1.81)	8.64 (±1.81)
***Absence of neutralizing antibodies against variants, N (%)***				
- **Wild-Type**	1 (2.4%)	4 (8.7%)	4 (14.3%)	2 (6.1%)
- **Alpha**	2 (4.9%)	0 (0%)	0 (0%)	0 (0%)
- **Delta**	4 (9.8%)	1 (2.2%)	3 (10.7%)	1 (3.0%)
- **Gamma**	4 (9.8%)	4 (8.7%)	2 (7.1%)	2 (6.1%)
- **Omicron**	20 (48.8%)	20 (47.5%)	18 (64%)	15 (45.4%)

## Data Availability

The dataset generated during and/or analyzed during the current study is available from the corresponding author on reasonable request.
